# Predicting Difficulty in Laparoscopic Cholecystectomies: An Evaluation of the Labbad-Vivas Score and Its Correlation With the Parkland Grading Scale

**DOI:** 10.7759/cureus.56185

**Published:** 2024-03-14

**Authors:** Roselys Serrano-González, Yeisson Rivero, Adriana Hernandez-Velasquez, Tamara Rodriguez-Rugel, Georcimar Mendez-Meneses, Andrea Vidal-Gallardo, Emiliana Garcia-Sánchez, Gabriel Gonzalez-Quinde, Jackner Antigua-Herrera, Yanira Zelaya-Ochoa, Marialejandra Paz-Castillo

**Affiliations:** 1 Department of Surgery, Universidad de Oriente Núcleo Anzoátegui, Barcelona, VEN; 2 Department of Surgery, Universidad Católica de Santiago de Guayaquil, Guayaquil, ECU; 3 Medico Cirujano, Universidad de los Andes, Merida, VEN; 4 Department of Surgery, Hospital Leon Becerra Camacho, Milagro, ECU; 5 Department of Surgery, Instituto Tecnológico de Santo Domingo, Santo Domingo, DOM; 6 Department of Surgery, Universidad de El Salvador, San Salvador, SLV

**Keywords:** labbad-vivas score, laparoscopic cholecystectomy, difficult cholecystectomy, parkland grading scale, predictive score

## Abstract

Background

Difficult laparoscopic cholecystectomy (DLC) denotes the surgical extraction of the gallbladder under circumstances where associated conditions within the same organ, adjacent structures, or patient-specific conditions impede a smooth, expeditious, and comfortable dissection. It is imperative to utilize tools that aid in anticipating this challenging surgical scenario, enabling the implementation of appropriate measures.

Objective

This study aimed to assess the effectiveness of the Labbad-Vivas score (LVS) in predicting DLC and its correlation with the Parkland Grading Scale (PGS).

Methodology

A prospective study was conducted, including patients diagnosed with gallstone disease undergoing LC (laparoscopic cholecystectomy) at the "Dr. Luis Razetti" University Hospital in Barcelona, Venezuela, between September and December 2023.

Results

Forty patients were studied, with 80% (n=32) being female and 95% (n=38) under the age of 65; surgeries were elective in 72.5% (n=29) of cases; 35% (n=14) had an LVS ≥16 (difficult cholecystectomy); and 62.5% (n=25) of patients presented Grades 1 and 2 on the PGS. Total cholecystectomy was performed in 95% (n=38) of the patients. The LVS showed a sensitivity of 80%, specificity of 92%, positive predictive value of 85.7%, and negative predictive value of 88.5% to predict DLC, with an area under the receiver operating characteristic curve of 0.897 (95% confidence interval (CI) = 0.792-1.003). A Pearson correlation coefficient of 0.805 (95% CI = 0.656 - 0.904) was obtained between both scores.

Conclusion

The use of the LVS score in the preoperative setting is feasible as a predictor of DLC, given its effectiveness and high correlation with the PGS.

## Introduction

Cholelithiasis is a disease characterized by the presence or formation of stones in the gallbladder. It constitutes one of the most frequent disorders of the digestive system in primary care, with cholecystectomy being the gold standard for its resolution [1]. In the United States, approximately 14 million women and 6 million men with an age range of 20 to 74 years have gallstones. The prevalence increases as a person ages. Obesity increases the likelihood of gallstones, especially in women, due to increases in the biliary secretion of cholesterol [2]. The prevalence of this disease is higher in Western countries; however, in Venezuela, there is no exact record of this data [3].

Cholelitaisis is asymptomatic in most cases, with stones discovered incidentally. Symptomatic patients present with right upper abdominal pain after eating greasy or spicy food, nausea, vomiting, and pain in the epigastrium that radiates to the right scapula or mid-back. Less than 50% of patients who have gallstones will develop symptoms. Complications from gallstones may include gallbladder inflammation leading to cholecystitis, common bile duct blockage resulting in bile duct infection and jaundice, or pancreatic duct blockage, which can cause pancreatitis [4].

Laparoscopic cholecystectomy (LC) is a minimally invasive surgical procedure for the removal of a diseased gallbladder. This technique has essentially replaced the open technique for routine cholecystectomies since the early 1990s [5]. At this time, LC is indicated for the treatment of cholecystitis (acute/chronic), symptomatic cholelithiasis, biliary dyskinesia, acalculous cholecystitis, gallstone pancreatitis, and gallbladder masses/polyps [6].

Difficult laparoscopic cholecystectomy (DLC) denotes the surgical extraction of the gallbladder under circumstances where associated conditions within the same organ, adjacent structures, or patient-specific conditions impede a smooth, expeditious, and comfortable dissection of the gallbladder. This results in prolonged surgical duration and heightened complications for the patient. Performing DLC demands heightened surgical expertise and necessitates critical decision-making distinct from conventional methods to mitigate risks for the patient [7]. Consequently, it becomes imperative to utilize tools that aid in anticipating this challenging surgical scenario, enabling the implementation of appropriate measures. Various international studies have proposed preoperative scoring systems to address this issue [8]. Nonetheless, it is crucial to devise a scale tailored to the idiosyncrasies of each population to streamline the efforts of surgeons and enhance the overall quality of medical and surgical care.

In Venezuela, between August 2018 and August 2019, Labbad and Vivas designed and applied a preoperative score for predicting DLC in the Surgery Service of the General Hospital "Dr. Domingo Luciani" in Caracas. The score was based on information derived from the physical examination, personal and surgical history, associated diseases, laboratory tests, and abdominal ultrasound findings. The main objective of this predictive score was to establish the appropriate diagnosis and develop an adequate work plan for the correct resolution of the clinical case based on a sample of 99 patients [9]. In this score, 12 parameters were evaluated: seven sociodemographic and clinical, such as sex, age, diabetes mellitus (DM), body mass index (BMI), history of biliary colic, history of surgery in the upper abdomen, and the presence of a palpable gallbladder; and five paraclinical, such as leukocytosis, gallstone size, impacted calculus in the infundibulum, pericholecystic collection, and cirrhosis. They defined difficult cholecystectomy as those cases with a score greater than or equal to 16. The predictive score made it possible to predict the risk of complications in a DLC based on the clinical and paraclinical characteristics of the patient at the time of evaluation. In addition, a statistically significant association was found between the presence of DLC, according to intraoperative findings, and the outcome of the experimental predictive score [9].

Intraoperative findings at the time of cholecystectomy vary according to the clinical presentation and may lead to a range of operative challenges. The prediction of the difficulty encountered during the procedure can offer the surgeon a range of benefits, including surgical planning, informing the patient, and predicting certain outcomes, such as the potential for conversion to open surgery [7].

The Parkland grading scale (PGS) for cholecystitis is a five-level rating system based on anatomical considerations and intraoperative inflammatory changes, known for its ease of implementation. The primary characteristic of this classification system is its capability to assess the severity level at the outset of surgery, providing a valuable tool for modifying the initial surgical strategy to achieve accurate and reliable stratification [10]. For instance, in cases where, despite adequate dissection, identification of anatomical structures and the critical view of safety prove challenging, a subtotal LC may be employed. This procedure boasts low morbidity and leverages the well-established advantages of minimally invasive surgery [11]. The PGS has undergone extensive validation and has demonstrated significant associations with the diagnosis of acute cholecystitis, surgical complexity, incidence of subtotal and open cholecystectomy rates, preoperative leukocytosis, duration of operation, and biliary leak rates, all of which increase in correlation with the escalating degrees of the scale [12].

Given the importance and benefits of anticipating a DLC, determining the best surgical technique, and reducing the probability of complications, it is proposed to evaluate the usefulness of the Labbad-Vivas score (LVS) while determining the correlation of this score with the degree of inflammation of the gallbladder intraoperatively with the PGS.

## Materials and methods

A prospective simple cohort study was conducted at the "Dr. Luis Razetti" University Hospital in Barcelona, Venezuela, focusing on patients from the general surgery department admitted with a diagnosis of gallstones between September and December 2023. The study included patients aged between 18 and 80 years who required surgical intervention, encompassing procedures such as laparoscopic total and subtotal cholecystectomy, conversion to open surgery, or intraoperative cholangiography. The exclusion criteria comprised pregnant patients and those with an oncological etiology. The sample selection was non-probabilistic by convenience, including all patients who met the aforementioned criteria and were admitted to the hospital during the specified period.

For each included patient, an assessment was conducted for each item on the Labbad-Vivas scale [9]. The punctuation assigned for each item was as indicated in Table 1.

**Table 1:** Predicting score for difficult laparoscopy according to Labbad and Vivas, 2023 [9]. HAS: history of abdominal surgery, BMI: body mass index, ERCP: endoscopic retrograde cholangiopancreatography.

Item	Category	Score
Sex	Female	1
	Male	2
Age group	<65 years	1
	>65 years	2
Diabetes mellitus	No	1
	Yes	2
BMI (kg/m2)	<30	1
	≥30	2
History of biliary colic, cholecystitis, choledocholithiasis, pancreatitis or ERCP.	No	1
	Yes	3
History of HAS	No	1
	Yes	2
Leukocytosis (mm3)	< 15.000	1
	≥ 15.000	2
Gallbladder wall thickness	< 4mm	1
	≥ 4mm	2
Palpable gallbladder	No	1
	Yes	2
Impacted stone in the infundibulum	No	1
	Yes	2
Pericholecystic collection	No	1
	Yes	2
Cirrhosis	No	3
	Yes	2

Demographic data were collected through the medical history and direct patient interviews. Ultrasound characteristics were determined based on the study performed by the attending physician on duty, holding a diploma in abdominal ultrasound. Laboratory results were obtained from studies conducted either in-house or externally at the center.

Additionally, information related to the type of surgery (emergency vs. elective), the specific surgical technique employed (total vs. subtotal cholecystectomy), and intraoperative findings based on the PGS were recorded. The assessment leading to the specific grade determination within the PGS was defined by the lead surgeon for each procedure based on Table 2.

**Table 2:** Parkland grading scale for cholecystitis [12].

Grade	Intraoperative Features
1	Normal-appearing gallbladder ('robin's egg blue'). No adhesions present. Completely normal gallbladder.
2	Minor adhesions at the neck, otherwise normal gallbladder. Adhesions restricted to the neck or lower part of the gallbladder
3	Presence of ANY of the following: Hyperemia, pericholecystic fluid, adhesions to the body, distended gallbladder
4	Presence of ANY of the following: Adhesions obscuring majority of gallbladder. Grade 1-3 with abnormal liver anatomy, intrahepatic gallbladder, or impacted stone (Mirrizi).
5	Presence of ANY of the following: Perforation, necrosis, inability to visualize the gallbladder due to adhesions.

Data were presented as mean and standard deviation for quantitative variables and as percentages for qualitative variables. The association between qualitative variables was assessed using the chi-square test and the exact Mid-P test. The Pearson index was employed to evaluate the correlation between quantitative variables, specifically the LVS and PGS. An analysis of the receiver operating characteristic (ROC) curve was conducted to assess the efficacy of the LVS. A p-value of <0.05 was considered statistically significant. For statistical analysis and reporting of results, IBM Corp. Released 2021. IBM SPSS Statistics for Windows, Version 28.0. Armonk, NY: IBM Corp. and the OpenEpi version 3.01 online tool were utilized.

A linear regression analysis was conducted to derive the regression line equation, facilitating the prediction of PGS values based on LVS values. The obtained equation serves as a predictive model for estimating PGS values from given LVS values.

To enhance the ease of LVS calculations and subsequently predict the likely PGS values, a web application was designed using Google Sheets. This application has been made accessible to the public through a shared link on Google Drive. Users can utilize this tool to input LVS values and obtain corresponding predicted PGS values, streamlining the process for practical application and decision-making in clinical settings.

## Results

During the study period, 260 patients diagnosed with gallstones were admitted to the surgery department, and 40 of them were ultimately included in the analysis. Among these, 80% (n=32) were female, with a mean age of 45.6 years and a standard deviation (SD) of 16.15. Additionally, 72.5% (n=29) of the surgical interventions were elective. Table 3 presents the distribution of patients by sex and age group, categorized by the type of surgery (elective vs. emergency). No statistically significant association was identified between these variables.

**Table 3:** Demographic features based on type of surgery among patients with cholecystopathies. a. Qualitative variables are expressed as number (%) *Chi-square test † Mid-P exact test

Features ^a^	Type of surgery		P-value
	Elective (n=29)	Emergency (n=11)	
Sex	-	-	-
Male	4(13.8)	4(36.4)	0.111 *
Female	25(86.2)	7(63.4)	
Age group	-	-	-
< 65 years	27(93.1)	11(100)	0.5205 †
≥ 65 years	2(6.9)	-	

The average Labbad-Vivas score was 15.32, with a standard deviation of 1.98. A total of 65% (n=26) of the patients had a score of less than 16, which is considered the threshold for indicating difficult cholecystectomy. Table 4 displays the frequencies observed for each of the LVS items. The items "BMI ≥30 cm" and "History of biliary colic, cholecystitis, choledocholithiasis, pancreatitis, or ERCP" were the most prevalent in the sample, occurring in 72.5% (n=29) and 75.5% (n=31) of cases, respectively.

**Table 4:** Frequency of LVS items in patients with cholecystopathies. LVS: Labbad-Vivas score, BMI: body mass index, HAS: high abdomen surgery, ERCP: endoscopic retrograde cholangiopancreatography.

Sociodemographic and clinical Items	N (%)	Paraclinical Items	N (%)
Sex	-	Leukocytosis (mm3)	-
Female	32(80)	< 15.000	34(85)
Male	8(20)	≥ 15.000	6(15)
Age group	-	Gallbladder wall thickness	-
<65 years	38(95)	< 4mm	34(85)
>65 years	2(5)	≥ 4mm	6(15)
Diabetes Mellitus	-	Impacted stone in the infundibulum	-
No	33(82.5)	No	37(92.5)
Yes	7(17.5)	Yes	3(7.5)
BMI (kg/m2)	-	Pericholecystic Collection	-
<30	11(27.5)	No	40(100)
≥30	29(72.5)	Yes	-
History of biliary colic, cholecystitis, choledocholithiasis, pancreatitis or ERCP.	-	Cirrhosis	-
No	9(22.5)	No	39(97.5)
Yes	31(75.5)	Yes	1(2.5)
History of HAS	-	-	-
No	39(97.5)	-	-
Yes	1(2.5)	-	-
Palpable Gallbladder	-	-	-
No	34(85)	-	-
Yes	6(15)	-	-

The Parkland grading scale was applied to the patient group, resulting in an average score of 2.34 and a standard deviation of 0.8. Concerning the specific grades of the scale, 62.5% (n=25) of patients were classified as grade 1 and 2. This classification indicates either no inflammation (grade 1) or minor inflammation changes (grade 2) in the gallbladder, aligning with the observation of non-difficult cholecystectomy findings in the operating room. All patients underwent a laparoscopic approach. The chosen technique for 97.5% (n=39) of the patients was total cholecystectomy, with only one patient managed through subtotal cholecystectomy.

**Figure 1:**
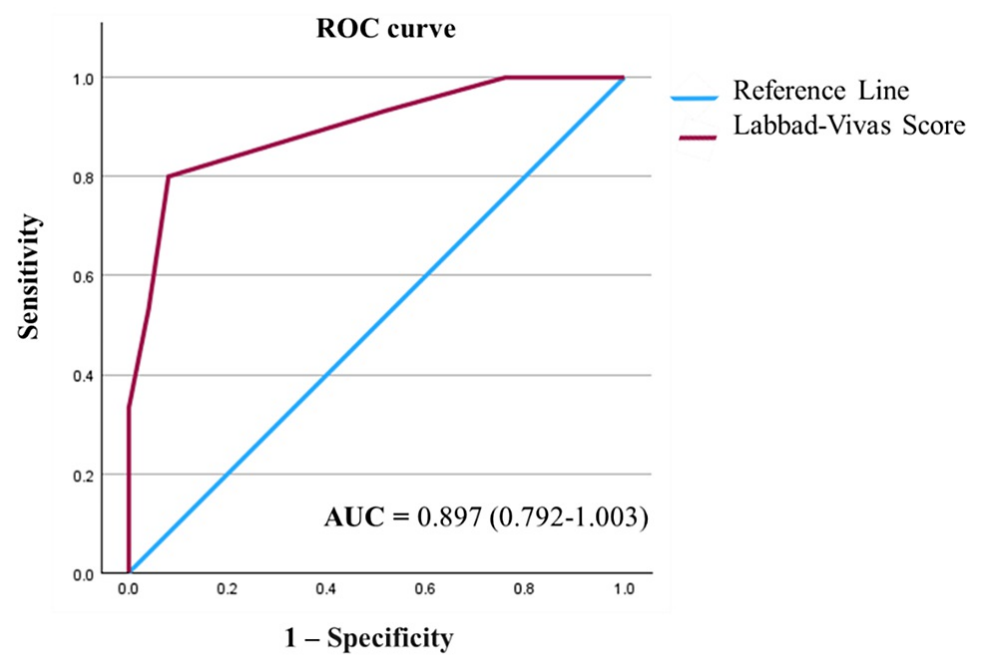
Receiver operating characteristic (ROC) curve for prediction of difficult laparoscopic cholecystectomy based on the Labbad-Vivas score. AUC: area under de curve

The association between the two scores was assessed through various approaches. A statistically significant relationship was observed between both scores based on the type of LC, whether difficult or not, with a p-value <0.001. Furthermore, it was determined that the LVS, compared to the PGS, exhibited a sensitivity of 80%, a specificity of 92%, a positive predictive value (PPV) of 85.7%, and a negative predictive value (NPV) of 88.5% for predicting DLC. The area under the ROC curve was 0.897 (95% confidence interval (CI) = 0.792-1.003, p <0.001), indicating a robust discriminative capacity of the LVS between the two categories of LC (difficult vs. not difficult). Figure 1 illustrates the ROC curve. 

**Figure 2:**
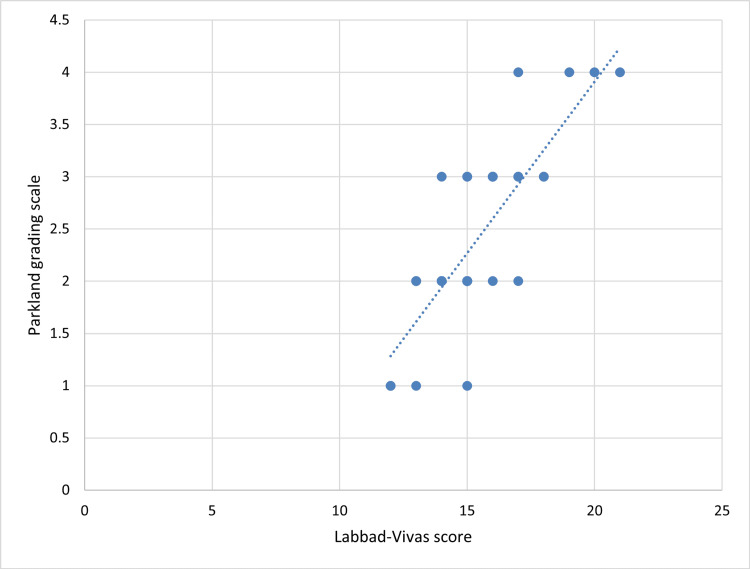
Scatter plot for the correlation between the Labbad-Vivas score and the Parkland grading scale.

Furthermore, the correlation between both scores was evaluated, obtaining a Pearson index of 0.805 (95% CI = 0.656-0.904), indicating a strong and statistically significant correlation between both variables. Figure 2 shows the scatter plot for this correlation, indicating that an increase in the LVS is directly proportional to an increase in the PGS.

Additionally, the simple linear regression formula utilized to predict PGS values based on LVS values is expressed as follows: y = 0.3281x - 2.6531. Here, "y" represents the dependent variable (PGS), and "x" represents the independent variable (LVS).

Based on the previous linear regression formula, we have designed a web application presented in the following link, through which readers could enter the data for the 12 variables of the LVS to predict a DLC and an approximate of the PGS [9,10,25]. 

## Discussion

The present study aimed to determine the feasibility of a preoperative scoring system for predicting DLC known as the Labbad-Vivas score, designed by Venezuelan doctors and applied to Venezuelan patients, finding that there was a high correlation between that score and intraoperative findings defined by the Parkland grading scale.

The general characteristics of the sample studied are consistent with other studies that included patients who underwent LC. The 2018 study by Tayeb et al., with 83 patients from a hospital in Pakistan who underwent LC, reported that most of their patients were women (61.5%) with an average age of 46 years [13], similar to the average age of our sample (45.6 years) and with female being the most common sex (80%). This predominance has been shown over the years in different studies [14] since women are more likely to have gallbladder diseases because estrogen increases biliary cholesterol secretion, causing cholesterol supersaturation of bile [15]. In Venezuela, the 2023 study by Núñez et al., with 61 patients from the University Hospital of Caracas, reported 67.19% of the patients under 60 years of age, being women in 85.2% of the cases [3]. In our study, the majority of patients were treated with elective surgeries (72.5%, n=29), similar to what was reported by Lucocq et al. in 2022 when they compared clinical outcomes between emergency vs. elective LC in 2768 patients, finding that 78.2% corresponded to the latter type [16]. Given the progressive nature of gallbladder diseases such as gallstones and cholecystitis in many cases, symptoms such as abdominal pain and nausea evolve, allowing the need for surgery to be identified and planned on a scheduled basis [4].

The 2023 study by Labbad-Labbad et al., conducted with 99 patients from the Hospital General del Este "Dr. Domingo Luciani" in Caracas, Venezuela, where the predictive LVS was proposed, reported that 47.5% of its patients were classified as having DLC [9]. In our sample of patients, a lower percentage (35%) of DLC was reported according to the same score, which can be explained by the difference in the features of both groups of patients since the population studied by Labbad-Labbad et al., compared to ours, reported a higher percentage of patients >65 years old (36.4 vs. 5%), with a prevalence of DM (22.2% vs. 17.5), with palpable gallbladder (49.5% vs. 15%), with leukocytes > 15000 (41.41 vs. 15), with wall thickness greater than 4 mm (51.5% vs. 15%), with pericholecystic collection (29.3% vs. 0), and with infundibulum stone (27.3 vs. 7.5). The only parameter that was higher in our sample was the percentage of patients with a BMI > 30 (72.5%, 38.4%). However, the study by Labbad-Labbad et al. does not indicate, for example, the percentage of patients who underwent emergency interventions, which would be interesting since it could explain the higher parameters of inflammation in their sample compared to ours [9].

Although the proposal by Labbad-Labbad et al. is the first of a validated score in Venezuelan patients, numerous studies with the same objective have been presented worldwide [9]. Some studies have focused on specific parameters, either ultrasound findings (gallbladder wall thickness, contracted gallbladder, impaction of gallstones at the neck of the gallbladder, and common bile duct stones) [17] or laboratory markers (C-reactive protein) [18]. Meanwhile, others, similar to LVS, have proposed a combination of different elements. Among the most recent is the study by Nassar et al. from 2019, which found that increasing age, the American Society of Anesthesiologists classification, male gender, diagnosis of common bile duct (CBD) stone or cholecystitis, thick-walled gallbladders, CBD dilation, use of pre-operative ERCP, and non-elective operations were found to be significant independent predictors of DLC when correlated to the Nassar operative difficulty scale (NODS) [7]. Other studies have also proposed predictive scoring systems for difficult cholecystectomies [8,19-21], considering similar criteria to those used in the LVS, such as male sex, history of abdomen surgery, palpable gallbladder, wall thickness≥4 mm, pericholecystic collection, or impacted stone.

Many of these studies have used their criteria (such as operative time or conversion rate to open cholecystectomy) to define DLC during surgery. Some have also used the NODS (originally proposed in 1995) [22]. In Venezuela, Núñez et al. [3] used this score to define intraoperative findings, but they also used the Parkland grading scale [10], finding that 82% of their patients had a score of I or II, considered a non-difficult LC, a percentage slightly higher than the 62.5% (n=25) reported in our patients for these categories. However, there are no other characteristics besides age or sex that allowed us to identify which factors may explain this difference.

In the 2022 study by Sisa-Segovia et al., studying 267 patients who underwent video LC in a hospital in Paraguay, they reported grades I and II in the PGS in 56.6% of the patients [23]. Sisa-Segovia et al. also reported similar features, such as elective surgeries in the majority of patients (68%) and leukocytosis only in 24.5% of patients, indicating serious inflammatory processes in a smaller proportion of patients, which may contribute to explaining the similarity the PGS reported. In addition, they performed total cholecystectomies in 95.13% of the cases, similar to the 97.5% (n=39) in our case [23].

In the Labbad-Labbad et al. study [9], although they indicate that all patients with the presence of difficult cholecystectomy, according to the findings of the predictive score, also had findings of difficult cholecystectomy intraoperatively, they did not make direct reference to the objective criteria for such classification in the operating room. On the other hand, the association between both classifications (preoperative vs. intraoperative) was categorical and not correlational, as proposed in this study.

The LVS had high values of sensitivity, specificity, PPV, NPV, and AUC to predict DLC, even higher than other predictive scores reported, such as the one presented by Ramírez-Giraldo et al. in 2022, after the analysis of 319 patients from a Colombian hospital. When comparing their predictor score with the NODS, the ROC curve showed an area of 0.88 under the curve, with a sensitivity, specificity, PPV, and NPV of 75.15%, 88.31%, 87.32, and 76.83%, respectively [24]. Even the preoperative risk score proposed by Nassar et al. in 2019 obtained an area under the ROC curve of 0.789 [7]. This suggests that the LVS not only performs well but could sometimes provide better results than other scores proposed internationally.

The conclusions of the present study must be interpreted within the framework of certain limitations, the main one being the small sample size from which the information could be extracted, which, in turn, influenced the fact that some of the variables had very low frequencies, as in the case of the type of surgical technique used, where only one of the patients underwent subtotal cholecystectomy, thus limiting the performance of further analyses. Therefore, it would be advisable to consistently record PGS or other validated intraoperative scores in all LC through future prospective multicenter studies, gathering a larger sample size. This approach will enhance the precision of results and contribute to the establishment of protocols for effective gallstone management. It is also important to highlight the strengths of the present study. It is the first to replicate and verify the effectiveness of the initial score designed by Venezuelan physicians based on data from Venezuelan patients to predict cases of difficult cholecystectomy with a high level of accuracy. Additionally, it is the first to digitize the application of a predictive score in the surgical field in the country.

## Conclusions

A noteworthy association between the Labbad-Vivas score and the Parkland grading scale suggests the potential of LVS as a reliable predictor for difficult cholecystectomy. It is recommended to incorporate the systematic application of LVS in the Venezuelan population to promptly identify patients at risk of challenging cholecystectomies. The findings of this study underscore the promising role of LVS as a valuable preoperative tool in the overall management of patients undergoing cholecystectomy.
